# Supplementary material for: Pyruvate Dehydrogenase Kinase 1 inhibition mediated oxidative phosphorylation enhancement in cartilage promotes osteoarthritis progression
Source: BMC Musculoskelet Disord. 2023 Jul 20;24:597. doi: 10.1186/s12891-023-06585-6 (PMC10357736; doi:10.1186/s12891-023-06585-6)
Supplement: Supplementary file 1 — Additional file 1. [file 12891_2023_6585_MOESM1_ESM.zip › MF.Gsea.1653627316242/GOMF_MRNA_3_UTR_BINDING.html]

Details for gene set GOMF\_MRNA\_3\_UTR\_BINDING[GSEA]

|  || Dataset | 55457\_collapsed\_to\_symbols.cls.cls #OA\_versus\_normal.cls.cls #OA\_versus\_normal\_repos |
| Phenotype | cls.cls#OA\_versus\_normal\_repos |
| Upregulated in class | normal |
| GeneSet | GOMF\_MRNA\_3\_UTR\_BINDING |
| Enrichment Score (ES) | -0.49801844 |
| Normalized Enrichment Score (NES) | -2.1007202 |
| Nominal p-value | 0.0 |
| FDR q-value | 0.008441864 |
| FWER p-Value | 0.016 |
Table: GSEA Results Summary

  

Fig 1: Enrichment plot: GOMF\_MRNA\_3\_UTR\_BINDING      
 Profile of the Running ES Score & Positions of GeneSet Members on the Rank Ordered List

  

| SYMBOL | TITLE | RANK IN GENE LIST | RANK METRIC SCORE | RUNNING ES | CORE ENRICHMENT || 1 | CRYZ | crystallin zeta [Source:HGNC Symbol;Acc:HGNC:2419] | 788 | 0.572 | -0.0409 | No |
| 2 | CARHSP1 | calcium regulated heat stable protein 1 [Source:HGNC Symbol;Acc:HGNC:17150] | 1498 | 0.429 | -0.0807 | No |
| 3 | NUDT21 | nudix hydrolase 21 [Source:HGNC Symbol;Acc:HGNC:13870] | 1734 | 0.393 | -0.0849 | No |
| 4 | RBM4 | RNA binding motif protein 4 [Source:HGNC Symbol;Acc:HGNC:9901] | 1970 | 0.364 | -0.0901 | No |
| 5 | NHP2 | NHP2 ribonucleoprotein [Source:HGNC Symbol;Acc:HGNC:14377] | 2026 | 0.358 | -0.0815 | No |
| 6 | ANGEL2 | angel homolog 2 [Source:HGNC Symbol;Acc:HGNC:30534] | 2608 | 0.290 | -0.1164 | No |
| 7 | APOBEC1 | apolipoprotein B mRNA editing enzyme catalytic subunit 1 [Source:HGNC Symbol;Acc:HGNC:604] | 2695 | 0.280 | -0.1130 | No |
| 8 | IGF2BP3 | insulin like growth factor 2 mRNA binding protein 3 [Source:HGNC Symbol;Acc:HGNC:28868] | 2770 | 0.273 | -0.1090 | No |
| 9 | FXR1 | FMR1 autosomal homolog 1 [Source:HGNC Symbol;Acc:HGNC:4023] | 3012 | 0.249 | -0.1188 | No |
| 10 | PARN | poly(A)-specific ribonuclease [Source:HGNC Symbol;Acc:HGNC:8609] | 3100 | 0.240 | -0.1170 | No |
| 11 | CIRBP | cold inducible RNA binding protein [Source:HGNC Symbol;Acc:HGNC:1982] | 3441 | 0.207 | -0.1360 | No |
| 12 | PCBP4 | poly(rC) binding protein 4 [Source:HGNC Symbol;Acc:HGNC:8652] | 4577 | 0.107 | -0.2207 | No |
| 13 | HNRNPD | heterogeneous nuclear ribonucleoprotein D [Source:HGNC Symbol;Acc:HGNC:5036] | 4830 | 0.085 | -0.2373 | No |
| 14 | DAZ1 | deleted in azoospermia 1 [Source:HGNC Symbol;Acc:HGNC:2682] | 4920 | 0.078 | -0.2414 | No |
| 15 | HNRNPA0 | heterogeneous nuclear ribonucleoprotein A0 [Source:HGNC Symbol;Acc:HGNC:5030] | 5485 | 0.033 | -0.2842 | No |
| 16 | TP53 | tumor protein p53 [Source:HGNC Symbol;Acc:HGNC:11998] | 5511 | 0.032 | -0.2850 | No |
| 17 | EXOSC4 | exosome component 4 [Source:HGNC Symbol;Acc:HGNC:18189] | 5693 | 0.016 | -0.2986 | No |
| 18 | AUH | AU RNA binding methylglutaconyl-CoA hydratase [Source:HGNC Symbol;Acc:HGNC:890] | 5911 | 0.001 | -0.3154 | No |
| 19 | RBFOX1 | RNA binding fox-1 homolog 1 [Source:HGNC Symbol;Acc:HGNC:18222] | 6152 | -0.016 | -0.3336 | No |
| 20 | DAZL | deleted in azoospermia like [Source:HGNC Symbol;Acc:HGNC:2685] | 6221 | -0.022 | -0.3381 | No |
| 21 | CPEB1 | cytoplasmic polyadenylation element binding protein 1 [Source:HGNC Symbol;Acc:HGNC:21744] | 6435 | -0.038 | -0.3533 | No |
| 22 | CPSF1 | cleavage and polyadenylation specific factor 1 [Source:HGNC Symbol;Acc:HGNC:2324] | 6629 | -0.052 | -0.3665 | No |
| 23 | RNF40 | ring finger protein 40 [Source:HGNC Symbol;Acc:HGNC:16867] | 6681 | -0.054 | -0.3685 | No |
| 24 | RBMS1 | RNA binding motif single stranded interacting protein 1 [Source:HGNC Symbol;Acc:HGNC:9907] | 7121 | -0.090 | -0.3995 | No |
| 25 | PUM2 | pumilio RNA binding family member 2 [Source:HGNC Symbol;Acc:HGNC:14958] | 7186 | -0.095 | -0.4011 | No |
| 26 | NOVA1 | NOVA alternative splicing regulator 1 [Source:HGNC Symbol;Acc:HGNC:7886] | 7394 | -0.110 | -0.4133 | No |
| 27 | ELAVL1 | ELAV like RNA binding protein 1 [Source:HGNC Symbol;Acc:HGNC:3312] | 7426 | -0.113 | -0.4116 | No |
| 28 | HNRNPC | heterogeneous nuclear ribonucleoprotein C [Source:HGNC Symbol;Acc:HGNC:5035] | 7437 | -0.114 | -0.4083 | No |
| 29 | MEX3D | mex-3 RNA binding family member D [Source:HGNC Symbol;Acc:HGNC:16734] | 7652 | -0.131 | -0.4203 | No |
| 30 | CCT5 | chaperonin containing TCP1 subunit 5 [Source:HGNC Symbol;Acc:HGNC:1618] | 7690 | -0.134 | -0.4184 | No |
| 31 | TARDBP | TAR DNA binding protein [Source:HGNC Symbol;Acc:HGNC:11571] | 7864 | -0.146 | -0.4266 | No |
| 32 | FMR1 | FMRP translational regulator 1 [Source:HGNC Symbol;Acc:HGNC:3775] | 7944 | -0.151 | -0.4274 | No |
| 33 | CPEB3 | cytoplasmic polyadenylation element binding protein 3 [Source:HGNC Symbol;Acc:HGNC:21746] | 8060 | -0.160 | -0.4306 | No |
| 34 | RNPS1 | RNA binding protein with serine rich domain 1 [Source:HGNC Symbol;Acc:HGNC:10080] | 8275 | -0.177 | -0.4409 | No |
| 35 | RSL1D1 | ribosomal L1 domain containing 1 [Source:HGNC Symbol;Acc:HGNC:24534] | 8286 | -0.177 | -0.4353 | No |
| 36 | CSDC2 | cold shock domain containing C2 [Source:HGNC Symbol;Acc:HGNC:30359] | 8294 | -0.178 | -0.4294 | No |
| 37 | RBMS2 | RNA binding motif single stranded interacting protein 2 [Source:HGNC Symbol;Acc:HGNC:9909] | 8373 | -0.184 | -0.4289 | No |
| 38 | EXOSC8 | exosome component 8 [Source:HGNC Symbol;Acc:HGNC:17035] | 8554 | -0.198 | -0.4358 | No |
| 39 | HNRNPA2B1 | heterogeneous nuclear ribonucleoprotein A2/B1 [Source:HGNC Symbol;Acc:HGNC:5033] | 8576 | -0.200 | -0.4303 | No |
| 40 | PABPC4 | poly(A) binding protein cytoplasmic 4 [Source:HGNC Symbol;Acc:HGNC:8557] | 8741 | -0.212 | -0.4354 | No |
| 41 | RBMS3 | RNA binding motif single stranded interacting protein 3 [Source:HGNC Symbol;Acc:HGNC:13427] | 8981 | -0.230 | -0.4458 | No |
| 42 | ZC3H12A | zinc finger CCCH-type containing 12A [Source:HGNC Symbol;Acc:HGNC:26259] | 9030 | -0.234 | -0.4411 | No |
| 43 | SERBP1 | SERPINE1 mRNA binding protein 1 [Source:HGNC Symbol;Acc:HGNC:17860] | 9238 | -0.251 | -0.4482 | No |
| 44 | ELAVL2 | ELAV like RNA binding protein 2 [Source:HGNC Symbol;Acc:HGNC:3313] | 9458 | -0.270 | -0.4556 | No |
| 45 | HNRNPU | heterogeneous nuclear ribonucleoprotein U [Source:HGNC Symbol;Acc:HGNC:5048] | 9847 | -0.306 | -0.4749 | No |
| 46 | PUM1 | pumilio RNA binding family member 1 [Source:HGNC Symbol;Acc:HGNC:14957] | 10145 | -0.335 | -0.4860 | Yes |
| 47 | RPL5 | ribosomal protein L5 [Source:HGNC Symbol;Acc:HGNC:10360] | 10201 | -0.341 | -0.4780 | Yes |
| 48 | ELAVL3 | ELAV like RNA binding protein 3 [Source:HGNC Symbol;Acc:HGNC:3314] | 10273 | -0.348 | -0.4710 | Yes |
| 49 | ELAVL4 | ELAV like RNA binding protein 4 [Source:HGNC Symbol;Acc:HGNC:3315] | 10412 | -0.362 | -0.4687 | Yes |
| 50 | ZFP36L2 | ZFP36 ring finger protein like 2 [Source:HGNC Symbol;Acc:HGNC:1108] | 10509 | -0.374 | -0.4627 | Yes |
| 51 | EXOSC9 | exosome component 9 [Source:HGNC Symbol;Acc:HGNC:9137] | 10606 | -0.384 | -0.4564 | Yes |
| 52 | RPS7 | ribosomal protein S7 [Source:HGNC Symbol;Acc:HGNC:10440] | 10608 | -0.384 | -0.4427 | Yes |
| 53 | TUT1 | "terminal uridylyl transferase 1, U6 snRNA-specific [Source:HGNC Symbol;Acc:HGNC:26184]" | 10699 | -0.393 | -0.4356 | Yes |
| 54 | CELF2 | CUGBP Elav-like family member 2 [Source:HGNC Symbol;Acc:HGNC:2550] | 10832 | -0.409 | -0.4311 | Yes |
| 55 | TIA1 | TIA1 cytotoxic granule associated RNA binding protein [Source:HGNC Symbol;Acc:HGNC:11802] | 10855 | -0.411 | -0.4181 | Yes |
| 56 | LARP1 | "La ribonucleoprotein 1, translational regulator [Source:HGNC Symbol;Acc:HGNC:29531]" | 10964 | -0.425 | -0.4112 | Yes |
| 57 | TIAL1 | TIA1 cytotoxic granule associated RNA binding protein like 1 [Source:HGNC Symbol;Acc:HGNC:11804] | 11049 | -0.438 | -0.4020 | Yes |
| 58 | KHSRP | KH-type splicing regulatory protein [Source:HGNC Symbol;Acc:HGNC:6316] | 11285 | -0.469 | -0.4034 | Yes |
| 59 | SECISBP2L | SECIS binding protein 2 like [Source:HGNC Symbol;Acc:HGNC:28997] | 11688 | -0.530 | -0.4157 | Yes |
| 60 | LARP4B | La ribonucleoprotein 4B [Source:HGNC Symbol;Acc:HGNC:28987] | 11695 | -0.531 | -0.3971 | Yes |
| 61 | LARP4 | La ribonucleoprotein 4 [Source:HGNC Symbol;Acc:HGNC:24320] | 11700 | -0.533 | -0.3782 | Yes |
| 62 | YBX3 | Y-box binding protein 3 [Source:HGNC Symbol;Acc:HGNC:2428] | 12024 | -0.599 | -0.3819 | Yes |
| 63 | FXR2 | FMR1 autosomal homolog 2 [Source:HGNC Symbol;Acc:HGNC:4024] | 12219 | -0.650 | -0.3736 | Yes |
| 64 | IGF2BP2 | insulin like growth factor 2 mRNA binding protein 2 [Source:HGNC Symbol;Acc:HGNC:28867] | 12244 | -0.657 | -0.3519 | Yes |
| 65 | PABPC3 | poly(A) binding protein cytoplasmic 3 [Source:HGNC Symbol;Acc:HGNC:8556] | 12257 | -0.663 | -0.3289 | Yes |
| 66 | DND1 | DND microRNA-mediated repression inhibitor 1 [Source:HGNC Symbol;Acc:HGNC:23799] | 12266 | -0.667 | -0.3056 | Yes |
| 67 | ILF3 | interleukin enhancer binding factor 3 [Source:HGNC Symbol;Acc:HGNC:6038] | 12353 | -0.692 | -0.2874 | Yes |
| 68 | CELF1 | CUGBP Elav-like family member 1 [Source:HGNC Symbol;Acc:HGNC:2549] | 12391 | -0.704 | -0.2649 | Yes |
| 69 | SECISBP2 | SECIS binding protein 2 [Source:HGNC Symbol;Acc:HGNC:30972] | 12416 | -0.712 | -0.2412 | Yes |
| 70 | PABPC1 | poly(A) binding protein cytoplasmic 1 [Source:HGNC Symbol;Acc:HGNC:8554] | 12562 | -0.778 | -0.2245 | Yes |
| 71 | RPL41 | ribosomal protein L41 [Source:HGNC Symbol;Acc:HGNC:10354] | 12674 | -0.847 | -0.2027 | Yes |
| 72 | DAZAP1 | DAZ associated protein 1 [Source:HGNC Symbol;Acc:HGNC:2683] | 12686 | -0.854 | -0.1729 | Yes |
| 73 | FUS | FUS RNA binding protein [Source:HGNC Symbol;Acc:HGNC:4010] | 12739 | -0.902 | -0.1445 | Yes |
| 74 | TAF15 | TATA-box binding protein associated factor 15 [Source:HGNC Symbol;Acc:HGNC:11547] | 12828 | -1.023 | -0.1145 | Yes |
| 75 | DDX5 | DEAD-box helicase 5 [Source:HGNC Symbol;Acc:HGNC:2746] | 12834 | -1.038 | -0.0776 | Yes |
| 76 | ZFP36 | ZFP36 ring finger protein [Source:HGNC Symbol;Acc:HGNC:12862] | 12846 | -1.052 | -0.0406 | Yes |
| 77 | ZFP36L1 | ZFP36 ring finger protein like 1 [Source:HGNC Symbol;Acc:HGNC:1107] | 12888 | -1.244 | 0.0009 | Yes |
Table: GSEA details [plain text format]

  

Fig 2: GOMF\_MRNA\_3\_UTR\_BINDING      
 Blue-Pink O' Gram in the Space of the Analyzed GeneSet

  

Fig 3: GOMF\_MRNA\_3\_UTR\_BINDING: Random ES distribution      
 Gene set null distribution of ES for **GOMF\_MRNA\_3\_UTR\_BINDING**
